# Empirical comparison of reduced representation bisulfite sequencing and Infinium BeadChip reproducibility and coverage of DNA methylation in humans

**DOI:** 10.1038/s41525-017-0012-9

**Published:** 2017-04-19

**Authors:** Juan J. Carmona, William P. Accomando, Alexandra M. Binder, John N. Hutchinson, Lorena Pantano, Benedetta Izzi, Allan C. Just, Xihong Lin, Joel Schwartz, Pantel S. Vokonas, Sami S. Amr, Andrea A. Baccarelli, Karin B. Michels

**Affiliations:** 1 Department of Environmental Health, Harvard T.H. Chan School of Public Health, Boston, MA 02115 USA; 2 Program in Quantitative Genomics, Harvard T.H. Chan School of Public Health, Boston, MA 02115 USA; 3 Center for Bioethics, Harvard Medical School, Boston, MA 02115 USA; 4 Department of Epidemiology, Harvard T.H. Chan School of Public Health, Boston, MA 02115 USA; 5 Obstetrics and Gynecology Epidemiology Center, Department of Obstetrics, Gynecology and Reproductive Biology, Brigham and Women’s Hospital, Harvard Medical School, Boston, MA 02115 USA; 6 Department of Biostatistics, Harvard T. H. Chan School of Public Health, Boston, MA 02115 USA; 7 Institute for Prevention and Cancer Epidemiology, Freiburg Medical Center, University of Freiburg, Freiburg, Germany; 8 Veterans Affairs Normative Aging Study, Veterans Affairs Boston Healthcare System, Department of Medicine, Boston University School of Medicine, Boston, MA 02118 USA; 9 Department of Pathology, Harvard Medical School, Brigham and Women’s Hospital, Boston, MA 02115 USA; 10 Translational Genomics Core, Partners Healthcare Personalized Medicine, Cambridge, MA 02139 USA

## Abstract

We empirically examined the strengths and weaknesses of two human genome-wide DNA methylation platforms: rapid multiplexed reduced representation bisulfite sequencing and Illumina’s Infinium BeadChip. Rapid multiplexed reduced representation bisulfite sequencing required less input DNA, offered more flexibility in coverage, and interrogated more CpG loci at a higher regional density. The Infinium covered slightly more protein coding, cancer-associated and mitochondrial-related genes, both platforms covered all known imprinting clusters, and rapid multiplexed reduced representation bisulfite sequencing covered more microRNA genes than the HumanMethylation450, but fewer than the MethylationEPIC. Rapid multiplexed reduced representation bisulfite sequencing did not always interrogate exactly the same CpG loci, but genomic tiling improved overlap between different libraries. Reproducibility of rapid multiplexed reduced representation bisulfite sequencing and concordance between the platforms increased with CpG density. Only rapid multiplexed reduced representation bisulfite sequencing could genotype samples and measure allele-specific methylation, and we confirmed that Infinium measurements are influenced by nearby single-nucleotide polymorphisms. The respective strengths and weaknesses of these two genome-wide DNA methylation platforms need to be considered when conducting human epigenetic studies.

## Epigenetics: choose your DNA methylation probing tool wisely!

Researchers who study human epigenetics need to carefully consider the platform used to measure genome-wide patterns of DNA methylation. A team led by Karin Michels and Andrea Baccarelli from Harvard University in Boston, Massachusetts, USA, empirically examined the strengths and weaknesses of two methylation profiling tools: Illumina’s Infinium BeadChip, which uses a microarray system to interrogate hundreds of thousands of methylation sites across the genome at single-nucleotide resolution; and a high-throughput sequencing-based approach known as rapid multiplexed reduced representation bisulfite sequencing, or rmRRBS. The former did a better job at reading methylation in protein-coding and mitochondrial-related genes, while the latter required less input DNA and covered more methylation sites across the genome. The authors conclude that a scientist’s platform preference should depend on the nature of his or her investigation.

## Introduction

Epigenetics is the study of mitotically and/or meiotically heritable gene regulation that is not due to changes in the primary sequence of DNA nucleotides.^1^ DNA methylation (DNAm) at cytosine residues in cytosine-guanine (CpG) dinucleotides is one of the most studied epigenetic marks; it is relatively easy to measure, critical to the maintenance of cellular identity,^2, 3^ and related to chromatin conformation and transcriptional programming.^3–6^ CpG loci are statistically underrepresented in mammalian genomes, but they are often concentrated in regions known as CpG “islands”, and ~60% of known human gene promoters contain CpG islands.^7^ Genomic regions 2000 base-pairs (2 kb) to each side of a CpG island are CpG “shores,” with CpG “shelves” extending 2 kb beyond CpG shores, with the rest of the genome termed “open sea”. These four contexts form the CpG “resort”, and the concentration of CpG loci decreases from islands to the open sea.^8^ The context of interest may depend on the research question. For example, since DNAm within promoter CpG islands exhibits patterns established during cellular differentiation and passed down through cell lineages,^9^ scientists investigating cell-lineage-specific gene regulation and/or identifying epigenetic biomarkers of cell and tissue types may focus on promoter CpG islands.^10–13^ On the other hand, since DNAm in CpG shores and shelves is more responsive to external factors,^14–16^ scientists investigating environmental programming of the genome via DNAm and/or trying to determine whether DNAm mediates known associations between exposures and diseases may focus on CpG shores and shelves.

Approaches that measure DNAm are continually being developed and refined. Many probe- and sequencing-based DNAm quantification approaches take advantage of sodium bisulfite treatment, which converts unmethylated cytosines to uracil (becoming thymine after PCR amplification) without changing methylated cytosines, allowing quantification of DNAm via estimation of cytosine-to-thymine at known CpG loci.^17^ Probe-based detection employs site-specific probes that hybridize onto bisulfite-converted DNA at target CpG loci, resulting in fluorescent signals. Infinium BeadChip arrays from Illumina (San Diego, CA), including the HumanMethyation450 (450K), and MethylationEPIC (850K), measure DNAm at pre-defined CpG loci with a generally high level of reproducibility and reliability,^8, 18–20^ but share some limitations. The required input DNA of 500 ng–1 µg precludes the use of Infinium arrays for scarce/precious samples, such as micro-dissected cancer biopsies. The invariable (and limited) set of CpG loci on Infinium arrays were designed to capture RefSeq genes and promoter CpG islands, excluding other regions of biologically meaningful variation. Customizable DNAm array options, such as Illumina’s VeraCode GoldenGate Methylation Assay use older technology^21^ and only examine a small number of CpG loci (384 loci per array), which are restricted by probe chemistry. Newer Infinium arrays also have issues with dye-biases, different probe chemistries and positional effects that are known to influence results and must be corrected during data processing.^22–24^ Infinium 450K probe cross-reactivity and ambiguous mapping to multiple locations in the human genome affects ~140,000 out of 485,000 probes (29% of the array), potentially reducing the number of usable probes to ~345,000,^25, 26^ an issue that persists with the newly released 850K, which includes >90% of the 450K probes.

Sequencing-based approaches for measuring DNAm across the human genome have rapidly evolved over the last decade. Whole-genome bisulfite sequencing (WGBS) requires a very large amount of DNA, often 3 μg, to generate a large amount of data that is expensive to store, much of which is not useful due to a lack of variability and/or overlap between samples.^27–29^ To overcome these limitations, reduced representation bisulfite sequencing (RRBS) was developed, requiring less DNA (10–200 ng) and able to generate more meaningful data than WGBS.^30^ A modified form of generalized RRBS, multiplexed RRBS (mRRBS) improved feasibility for large studies by allowing multiple libraries per sequencing lane,^29^ and others have further modified mRRBS for particular applications.^31, 32^ We refined mRRBS to allow for faster and more efficient throughput, thus creating a “rapid multiplexed” RRBS (rmRRBS) platform for the quantification of genome-wide DNAm. Briefly, genomic DNA (gDNA) is digested with MspI restriction enzyme, which targets CpG-rich areas, to generate a library of fragments that each contains at least two CpG loci. After ligating indexed oligonucleotide adapters to these fragments and performing size selection using magnetic beads, rmRRBS libraries are pooled, treated with sodium bisulfite, PCR amplified, cleaned up, and then subjected to next-generation sequencing (NGS). The sequenced reads are then aligned to a reference genome and stacked to yield “read depth,” i.e., the number of reads per region (e.g., 1×, 2×, etc.). Unlike probe-based approaches, sequencing-based approaches like rmRRBS are able to measure single-nucleotide polymorphisms (SNPs) and quantify allele specific DNAm (ASM).^33–35^ SNPs can impact DNAm estimates by altering the primary sequence of nucleotides to eliminate (or add) a CpG locus, and by influencing DNAm at nearby CpG loci.^36^ ASM is of particular interest to studies of human imprinting, a phenomenon where one parental allele is expressed in a parent-of-origin-specific manner, while the other is silenced. For example, the 11p15 chromosomal region contains a contiguous multigene imprinting cluster including *H19*, a long non-coding RNA gene that is only expressed by the hypomethylated maternal allele, while the hypermethylated paternal allele is silenced.^37^ Loss of imprinting in 11p15 is associated with childhood growth disorders and cancer, such as Beckwith–Wiedemann syndrome and Wilms tumor,^38^ as well as adult cancers.^39^

This work is an in-depth examination of rmRRBS genomic coverage and precision for a range of human DNA input quantities. We also explore analytic approaches to RRBS data, including genomic tiling, SNP detection, and ASM quantification. Our goal is to inform researchers who are considering probe-based and/or sequencing-based genome-wide DNAm platforms for epigenetic investigations from basic science to epidemiologic studies.

## Results

### Genomic coverage

**Fig. 1:**
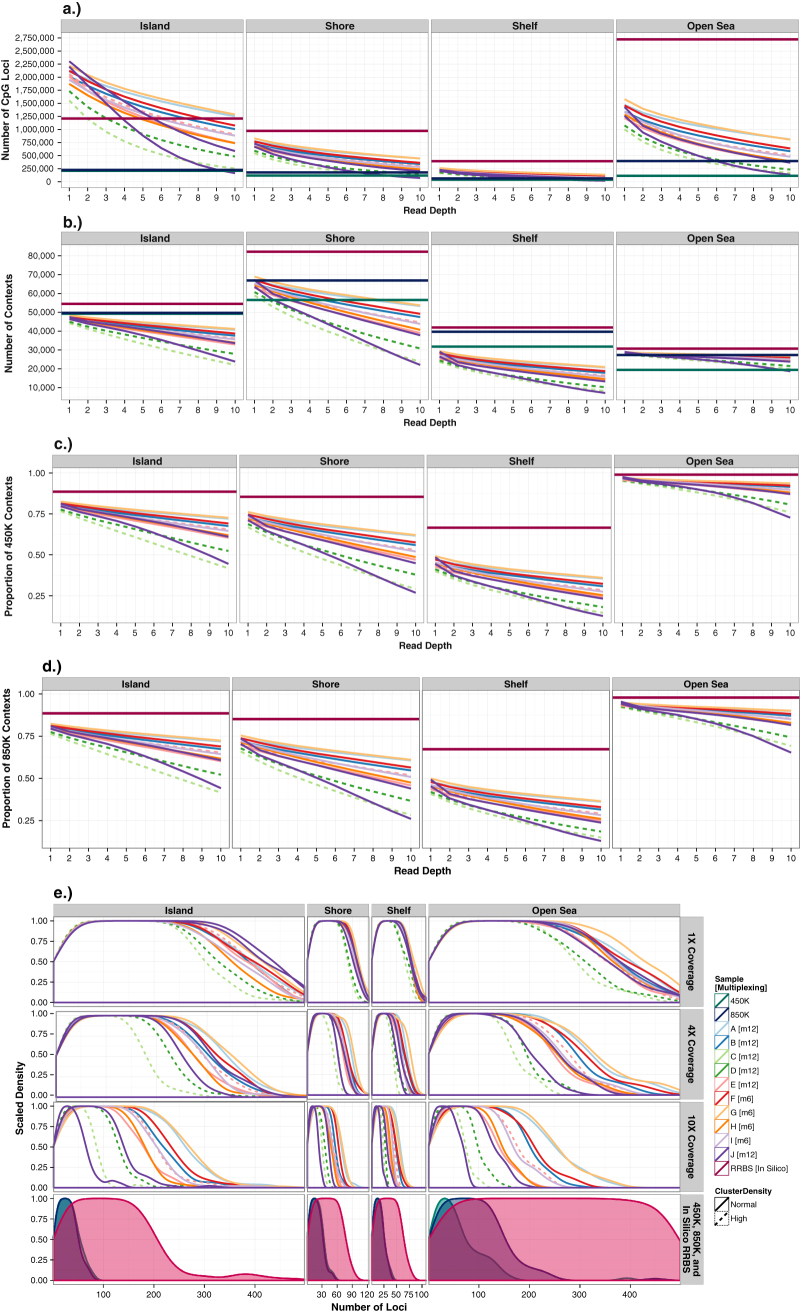
Genomic coverage of 12 rmRRBS libraries at different read depths stratified by CpG resort context. **a** The total number of CpG loci covered, **b** the number of discrete contexts in which at least one CpG locus is covered, **c** the proportion of exactly the same contexts from the Infinium HumanMethyaltion450 (450K) array for which at least once CpG locus was covered, and **d** the proportion of exactly the same contexts from the Infinium MethyaltionEPIC (850K) array for which at least once CpG locus is covered are plotted from ≥1× to ≥10× reads. In panels (**a**)–(**d**), the dark red horizontal lines indicate predicted RRBS genomic coverage, bioinformatically determined by performing RRBS in silico*.* In panels (**a**) and (**b**), the dark green horizontal lines indicate Infinium 450K genomic coverage, and the dark blue horizontal lines indicate Infinium 850K genomic coverage. (**e**) The distribution of the number of CpG loci measured in each discrete region that was covered is stratified in columns by type of CpG context and in rows by rmRRBS read depth, with CpG density distributions for in silico RRBS, the Infinium 450K, and the Infinium 850K plotted together in the fourth row. The peak of the density is the mode, and indicates most common number of CpG loci measured in each region. In all panels, unique individuals A through J appear as different colored lines where solid lines indicate normal cluster density and dotted lines indicated high cluster density

**Fig. 2:**
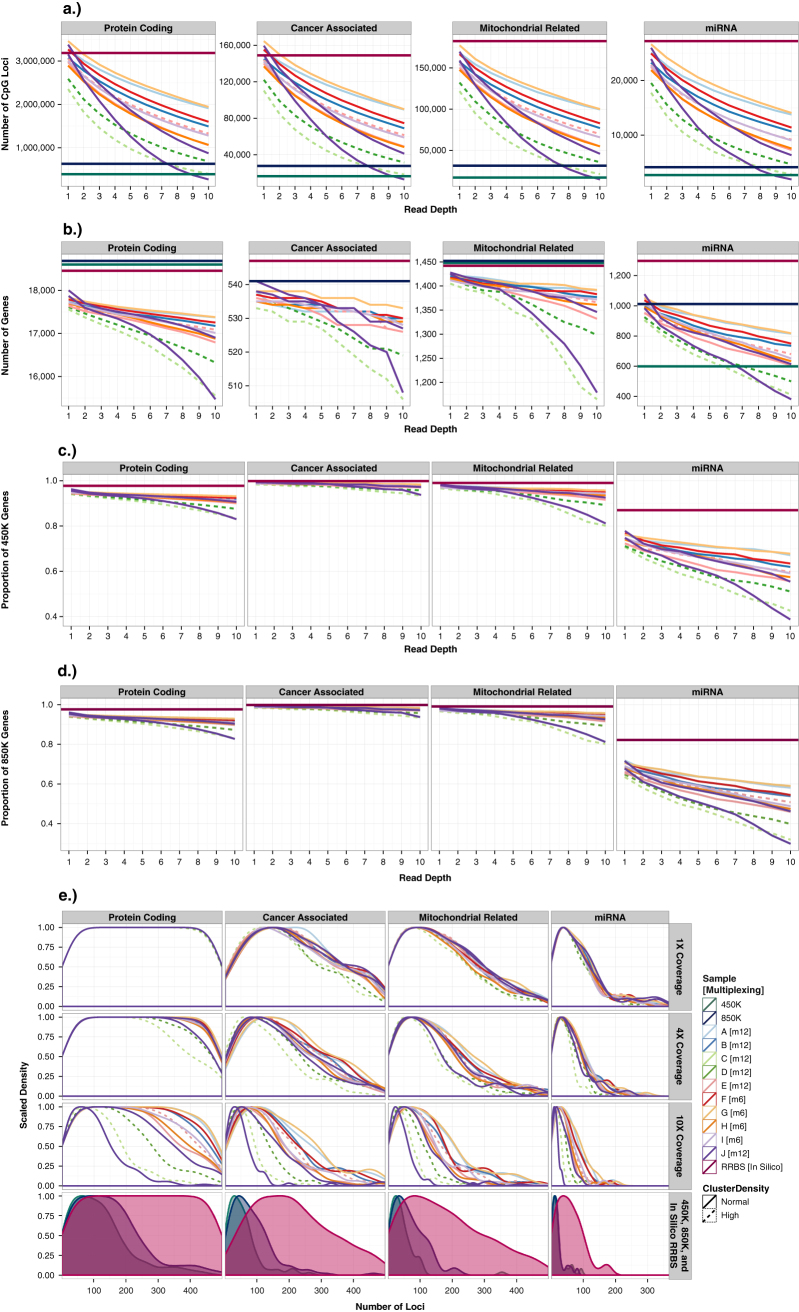
Genomic coverage of 12 rmRRBS libraries at different read depths stratified by four types of genes. **a** The total number of CpG loci covered **b** the number of genes in which at least one CpG locus is covered **c** the proportion of genes on the Infinium HumanMethyation450 (450K) array for which at least once CpG locus is covered, and **d** the proportion of genes on the Infinium MethyationEPIC (850K) array for which at least once CpG locus is covered are plotted from ≥1× to ≥10× reads. In panels (**a**)–(**d**), the dark red horizontal lines indicate predicted RRBS genomic coverage, bioinformatically determined by performing RRBS in silico. In panels (**a**) and (**b**), the dark green horizontal lines indicate Infinium 450K genomic coverage, and the dark blue horizontal lines indicate Infinium 850K genomic coverage. (**e**) The distribution of the number of loci measured in each gene that was covered is stratified in columns by type of gene and in rows by mRRBS read depth, with CpG density distributions for in silico RRBS, the Infinium 450K and the Infinium 850K plotted together in the fourth row. The peak of the density is the mode, and indicates most common number of CpG loci measured in each gene. In all panels, unique individuals A through J appear as different colored lines where solid lines indicate normal cluster density and dotted lines indicated high cluster density

We constructed and sequenced 86 rmRRBS libraries using human peripheral blood gDNA obtained from 10 adult males, labeled A–J (Supplementary Table S1), which we also evaluated via the Infinium 450K. We performed RRBS *in silico* to indicate expected RRBS coverage, and used Illumina’s manifest files to determine expected 450K and 850K coverage. We first selected 12 of the rmRRBS libraries—including two technical replicates—to examine in detail. Reflecting the rmRRBS enrichment protocol, genomic coverage varied by genetic element (Figs. 1 and 2; Supplementary Table S2). The number of reads at specific CpG loci for each of the 12 libraries can be viewed in the University of California Santa Cruz Genome Browser (see Supplemental Materials). Stratifying by CpG resort context, all 12 rmRRBS libraries covered from hundreds to over a million more CpG loci than the Infinium arrays at ≥4×, and five to ten libraries covered from hundreds to over a million more CpG loci than the Infinium arrays at ≥10× (Fig. 1a). This trend was recapitulated when we stratified by four categories of human genes: protein-coding genes, cancer-associated genes, nuclear-encoded genes related to mitochondrial function, and microRNA (miRNA) genes (Fig. 2a). With the exception of CpG islands and miRNA genes, our rmRRBS libraries did not perform as well as predicted by performing RRBS *in silico*.

Five libraries covered a greater diversity of discrete CpG shores than the 450K at ≥4×, eleven libraries covered more open sea regions than the 450K at ≥10×, but none covered as many CpG islands and shelves as the 450K, and the 850K covers at least as many of all four contexts as the rmRRBS libraries (Fig. 1b). At ≥4×, a majority of the rmRRBS libraries measured at least one CpG locus in ~75% and ~65% of the exact same CpG islands and shores, respectively, captured on the Infinium arrays, but only about a third of the exact same CpG shelves (Fig. 1c–d). Even at ≥10×, nine rmRRBS libraries covered a larger number of microRNA genes than the 450K and a comparable absolute number of cancer-associated genes, protein-coding genes, and nuclear-encoded genes with mitochondrial function to both the 450K and 850K (Fig. 2b). Moreover, overlap between the rmRRBS libraries at ≥10× and the Infinium arrays ranged from 83% to 93% for protein-coding genes, 93–98% for cancer-associated genes, 80–96% for mitochondrial-related genes, and 30–68% for microRNA genes (Fig. 2c–d). Regardless of genomic context, rmRRBS covered more CpG loci per region than the Infinium arrays (Figs. 1e, 2e). Likewise, rmRRBS measured a higher density of CpG loci in all 30 known human imprinting regions—which were covered at ≥10× by all 12 libraries—than the Infinium arrays (data not shown).

**Fig. 3:**
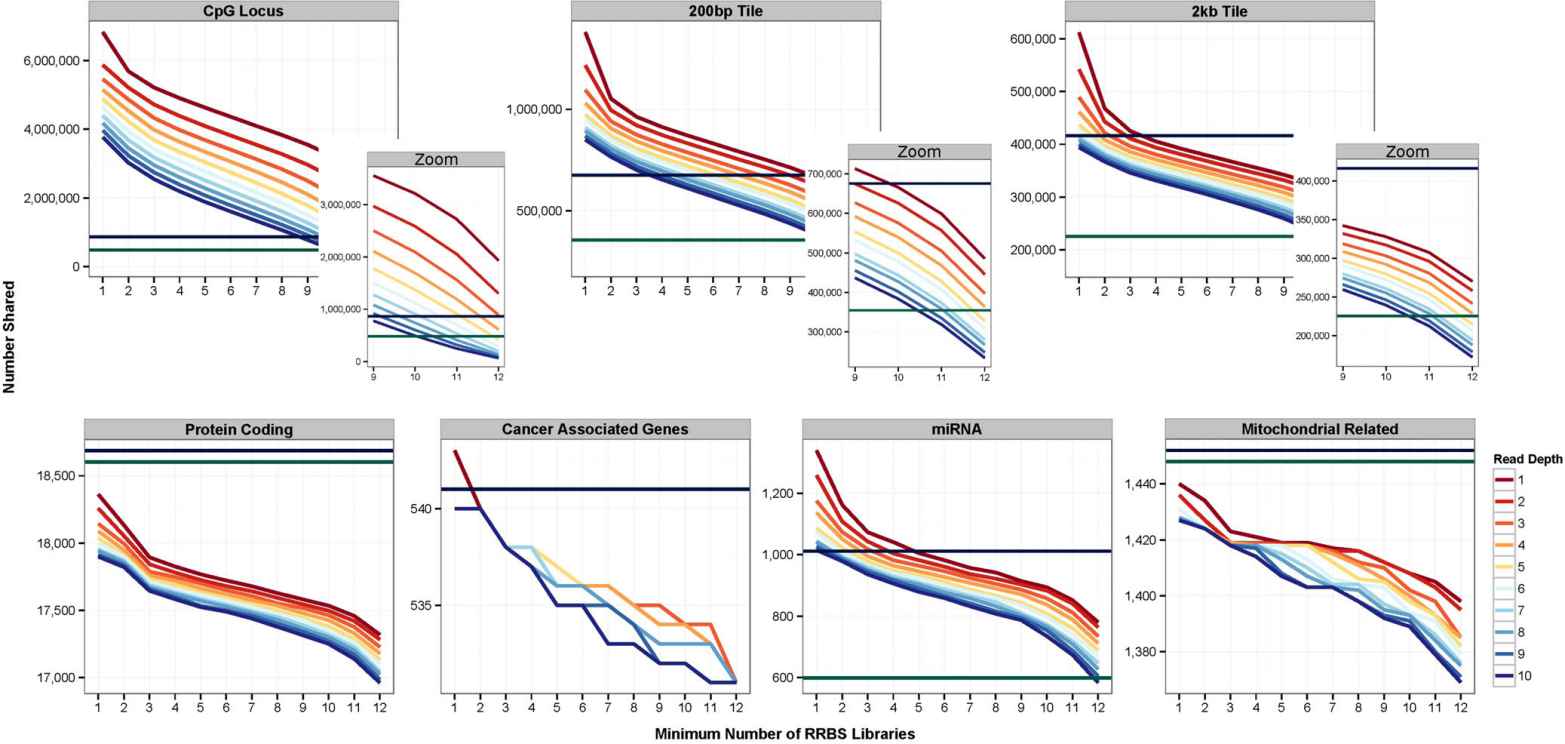
Overlap in genomic coverage between different rmRRBS libraries. The number of CpG loci, CpG resort context-restricted genomic tiles up to 200 base pairs (200 bp tiles) or 2000 base pairs (2 kb tiles) in length, protein coding genes, cancer-associated genes, microRNA genes, and nuclear encoded genes related to mitochondrial function are plotted relative to the number of rmRRBS libraries in which exact matches overlap. Different color lines correspond rmRRBS read depth, ranging from ≥1× to ≥10× reads per CpG locus, 200 bp, 2 kb tile, or gene. For reference, horizontal lines indicate the number of CpG loci, tiles or genes covered by the Infinium HumanMethylation450 (450K; dark green) and the Infinium MethyationEPIC (850K; dark blue)

We next investigated overlap in genomic coverage between rmRRBS libraries, which is dependent on both read depth and the minimum number of libraries that must capture the same CpG locus. In addition to looking at individual CpG loci, we also divided the human genome into discrete genomic “tiles” that never span more than one type of CpG resort context. As expected, the correlation between DNAm at CpG loci decreased as the distance between the loci increased. However, the rate at which this correlation dissipated was dependent on the CpG resort context. DNAm in CpG islands was highly correlated across much larger regions than other resort contexts (Supplementary Fig. S1). To appraise the impact of this variability across regions on reproducibility, we created two sets of genomic tiles to increase overlap across libraries: one set with a maximum size of 200 base-pairs (200 bp) and another set with a maximum size of 2000 base-pairs (2 kb), the previously defined size of a CpG shore or shelf region. All 12 libraries at ≥4× read depth, and up to 10 libraries at ≥10×, overlapped at more individual CpG loci (>482,421), 200 bp tiles (>354,806) and 2 kb tiles (>225,403) than are captured by the 450K (Fig. 3). Furthermore, we found that different rmRRBS libraries covered a large number of exactly the same protein-coding, cancer-associated, microRNA and mitochondrial-related genes. The best overlap in coverage among the types of genes considered was in microRNAs. In fact, all 12 rmRRBS libraries at ≥4×, and up to 10 libraries at ≥10×, overlapped at >715 microRNA genes—more than the 599 microRNA genes present on the 450K. Figure 3 also displays putative 850K coverage, which is particularly improved over the 450K for 200 bp tiles, 2 kb tiles and microRNA genes. The number of cancer-associated and mitochondrial-related genes that overlapped between rmRRBS libraries were ≥ 95% and ≥90%, respectively, of the number on the Infinium arrays.

### DNAm quantification

**Fig. 4:**
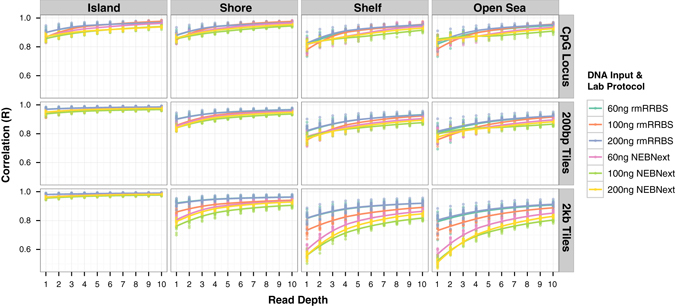
Reproducibility of rmRRBS DNA methylation measurements. The Pearson correlations between DNA methylation measurements for combined reads from all 12 libraries prepared under each of six different experimental conditions and reads from the individual 12 libraries prepared under that condition, stratified by CpG resort context, ranging from ≥1× to ≥10× reads for the individual library, are plotted for CpG loci, 200 base pair genomic tiles (200 bp tiles), and 2000 base pair genomic tiles (2 kb tiles)

**Fig. 5:**
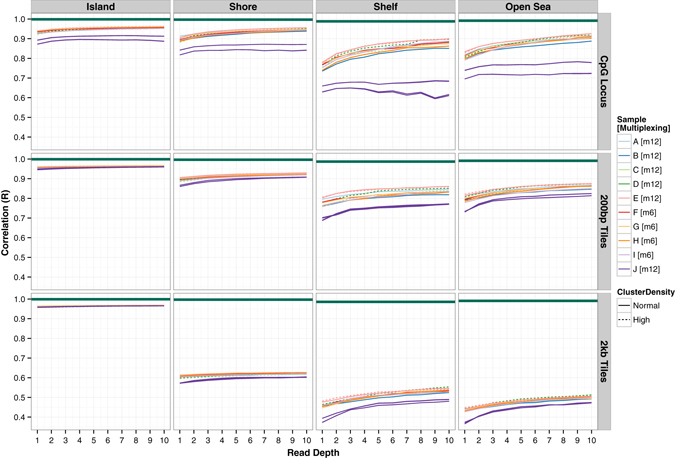
Concordance of rmRRBS and Infinium DNA methylation measurements. The Pearson correlations between DNA methylation measurements from 12 rmRRBS libraries derived from ten different participants’ (labeled A–J) gDNA and DNA methylation measurements for the corresponding Infinium HumanMethylation450 (450K) data derived from the same ten participants’ gDNA is plotted from ≥1× to ≥10× rmRRBS reads. Results are stratified by CpG resort context and shown for CpG loci, 200 base pair genomic tiles (200 bp tiles), and 2000 base pair genomic tiles (2 kb tiles)

In order to test the precision of rmRRBS DNAm measurements, and to determine the influence of experimental variables, we prepared 72 technical replicates from one individual’s gDNA sample—12 libraries each under six different conditions, including two laboratory protocols (NEBNext and rmRRBS) with 60, 100, and 200 ng of starting gDNA input (Supplementary Table S3). At 10x coverage and with 200 ng of starting gDNA, the rmRRBS protocol captured a greater number of CpG loci on average, specifically among CpG islands, shores, and shelves on average. However, the NEBNext protocol tended to capture more open sea sites (Supplementary Table S4). For each CpG locus, 200 bp tile or 2 kb tile, we combined all sequencing reads for each condition to serve as standards. Stratifying by CpG context, we calculated the correlation of DNAm between each library and its standard, revealing that the reproducibility of rmRRBS DNAm measurements increased with read depth and CpG density (Fig. 4). At ≥10x reads, R-values between libraries and their standards were >0.9 for islands and shores and >0.85 for shelves and open sea. Within CpG islands, reproducibility further improved when 200 bp and 2 kb tiles were employed. Outside of CpG islands, using 200 bp tiles had little effect, whereas using 2 kb tiles reduced reproducibility, particularly in shelves and open sea regions. Comparing DNAm measurements via rmRRBS to averaged triplicate Infinium measurements for the same gDNA sample, concordance between rmRRBS and Infinium DNAm measurements increased with both CpG density and rmRRBS read depth (Fig. 5). The Pearson correlation between rmRRBS estimated percent methylation and 450K methylation level for all overlapping loci at ≥1x and ≥10x can be viewed in Genome Browser (Supplemental Materials). For ten libraries at ≥10x reads, correlations with Infinium DNAm at individual CpG loci were >0.95, >0.93, >0.85, and >0.88 for islands, shores, shelves, and open sea, respectively. Using 200 bp tiles rather than matching individual CpG loci increased the correlation between platforms, regardless of CpG context. Although using 2 kb tiles increased the correlation between platforms for DNAm measured in CpG islands even more than using 200 bp tiles, it decreased the correlation between platforms outside of CpG islands.

**Fig. 6:**
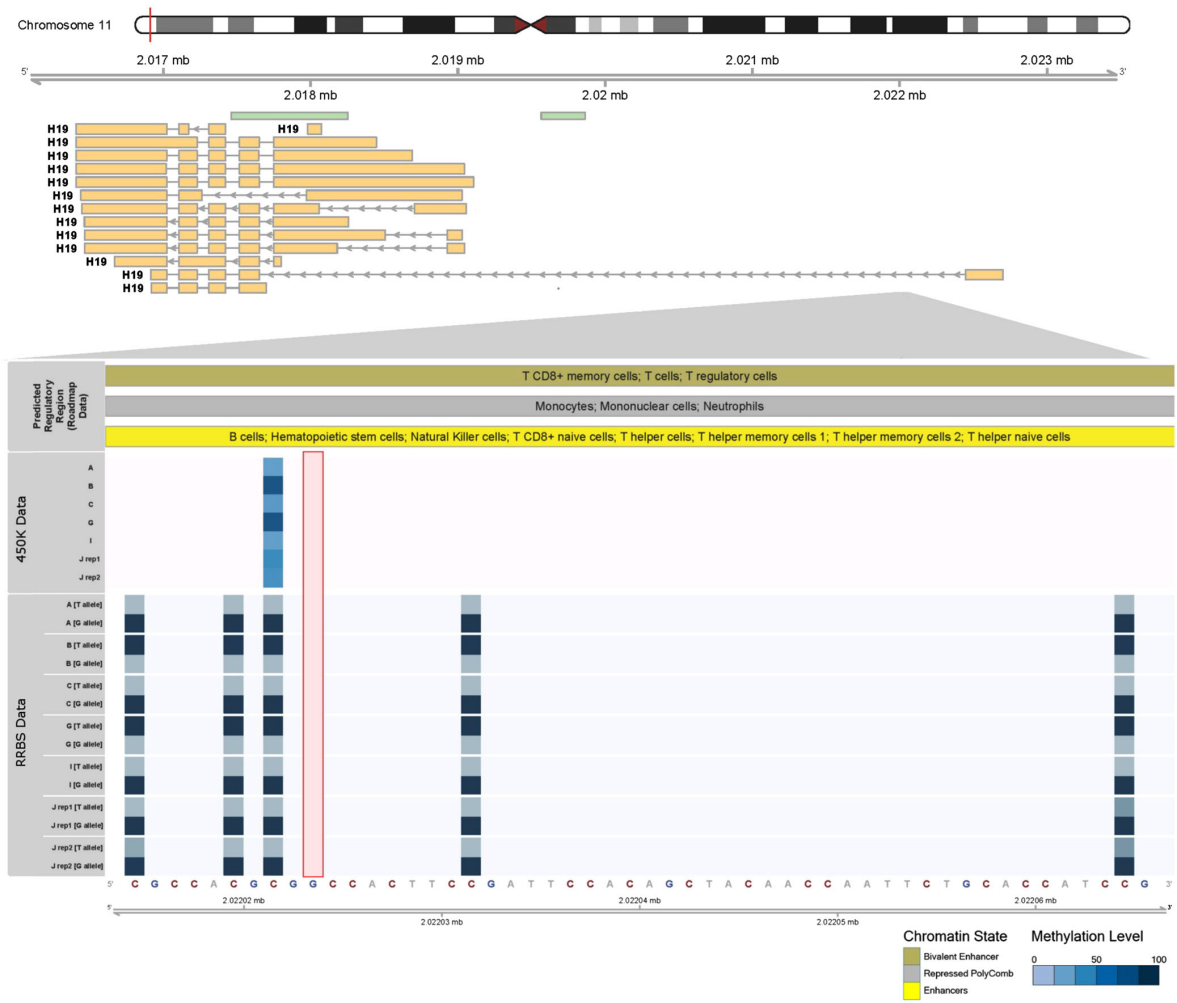
Genotype and allele-specific DNA methylation are measured by rmRRBS but not the Infinium BeadChip. A diagram illustrating the position of the target region in *H19* within the 11p15 imprinting region is shown at the top of the figure. The primary sequencing of DNA nucleotides appears at the bottom, with a G/T single nucleotide polymorphism (SNP) highlighted in a red box in the body of the figure. Monoallelic DNA methylation for the five CpG loci covered by rmRRBS, as well as overall DNA methylation for the single CpG locus covered by the Infinium HumanMethylation450 (450K) array, are shown for the six individuals (including one replicate of J) who were heterozygous for the G/T SNP with blue saturation of heatmap cells indicating DNA methylation levels. Predicted regulatory functions based on Epigenetic Roadmap chromatin state data is shown above the DNA methylation heatmaps

To assess the ability for rmRRBS to detect SNPs and measure ASM, we plotted ASM within the imprinting control region of *H19* in proximity to a common G/T SNP (rs10840167) for which seven rmRRBS libraries were heterozygous with ≥4x reads per allele. Epigenomic Roadmap data for the region with the SNP predicts leukocyte regulatory functions, including enhancer activity for lymphoid immune cells and repressed polycomb activity for myeloid immune cells.^40^ Across the five CpG loci captured on the same read as the SNP, we found that one allele was fully methylated and the other was fully unmethylated, suggesting that these loci exhibit parental imprinting (Fig. 6). Since the Infinium array cannot measure ASM, the expected methylation value for these loci is 50% (the average of DNAm from the two alleles). Instead the two samples that showed higher methylation on the T-allele had high (~89%) methylation at the locus that overlapped with this region on the Infinium 450K, whereas the methylation level ranged from 35 to 48% for the rest of the samples.

## Discussion

We empirically assessed a derivative of RRBS optimized for high-throughput, called rmRRBS, and Infinium BeadChip technology. Our work suggests that there are many trade-offs to consider when selecting between array-based and/or sequencing-based DNAm platforms for human research studies. Compared to the Infinium array, NGS-based rmRRBS is capable of covering more CpG loci and a greater abundance of CpG shores, which are thought to be important for exposure-related epigenetic modifications, as well as open sea regions. Additionally, rmRRBS tends to cover more CpG loci within a given region than the Infinium. Unlike array-based approaches, however, the exact same CpG loci are not always measured across rmRRBS libraries. Moreover, the number of reads covering each site impacts quantitative estimation of DNAm, thereby influencing reproducibility. All rmRRBS libraries are generated by enzymatic digestion of the genome, so there is no guarantee that a fragment will be generated even if the correct restriction sites flank the area. Furthermore, even if the digestion generates a particular fragment, it must also be properly ligated onto adapters, bisulfite converted, PCR amplified, sequenced, and successfully aligned in order for a CpG locus therein to be analyzed as a discrete read. Thus, there will usually be some experimental variation between libraries, which manifests as differences in genomic coverage and depth. This work considered variability due to DNA input quantity for a commonly used biologic sample: human blood. Future work should explore other potential sources of variability, particularly those affecting DNA quality, such as different origins (e.g., tumor tissue, cell lines, cultured primary cells), storage conditions, purities, and extraction methods. Since RRBS libraries are generated by fractionating DNA early in the procedure, premature fractionation is unlikely to significantly affect results. This suggests that rmRRBS is a suitable platform for samples of uncertain or poorer quality, such as DNA derived from formalin fixed paraffin embedded tissue and clinical samples that were processed or stored under sub-optimal conditions. However, additional systematic experiments are needed to assess rmRRBS performance relative to array-based approaches for poorer quality DNA.

Considering DNAm across tiles, rather than individual CpG loci, can improve overlap across rmRRBS libraries. While different libraries may not cover precisely the same CpG loci, they may cover CpG loci in close proximity. As anticipated, the impact of tile size on rmRRBS reproducibility and concordance with Infinium measurements depended on the variability of DNAm within the genomic region. For less variable elements, such as CpG islands, even relatively large tiles improved the precision of DNAm estimates. In regions where the correlation between loci dropped quickly with distance, such as shores and shelves, smaller tiles were necessary. Our capacity to estimate these regional DNAm patterns is facilitated by the greater density of CpG loci among contexts covered compared to the Infinium.

Unlike Infinium BeadChip arrays, rmRRBS allows for both genotyping (i.e., SNP detection) and the measurement of ASM, making it a powerful platform for studies of imprinting and other types of monoallelic DNAm. Our examination of the 11p15 imprinting region (specifically *H19*) not only revealed that rmRRBS is effective at measuring ASM, but it also confirmed that Infinium DNAm quantification can be confounded by the status of SNPs near the target CpG, thus introducing bias that is usually avoided by removing all known SNP proximal Infinium probes from analysis.

If sample material or DNA is scarce, it may not be possible to use Infinium BeadChip arrays, which require 500 ng–1 μg of DNA (or WGBS, which requires 3 μg of DNA). Herein, we demonstrate that rmRRBS can measure genome-wide DNAm using 60–200 ng of DNA. Both platforms require high-quality DNA input. There is a possibility that rmRRBS may be more sensitive to microbial contamination, such as might be found in saliva or buccal cell DNA, than the Infinium array, but this remains to be tested. The overall costs of running rmRRBS and the Infinium are comparable considering requisite labor, reagents, and bioinformatics. If the investigator has abundant DNA, Infinium arrays are likely the better option due to their consistency in both genomic coverage and DNAm estimates. There are some concerns involving dye-bias between the different probe types, as well as positional and batch effects on Infinium arrays, but these have been mostly resolved in well-established bioinformatics pipelines.^22, 24, 41, 42^ On the other hand, rmRRBS offers greater flexibility in the genomic regions that are detected, with the potential to investigate DNAm at sites that are not interrogated by Infinium arrays, which could lead to the discovery of novel biomarkers that would be missed otherwise. In our work, we found that a larger number (and a different pool) of microRNA genes were covered by rmRRBS than the Infinium 450K. This suggests that rmRRBS is of particular utility for researchers interested in studying DNAm in human microRNA genes. Furthermore, epigeneticists interested in SNP genotyping and/or allele-specific DNAm would benefit from employing rmRRBS in their research, and the method has the potential for clinical applications including diagnostics for imprinting disorders and the detection of diagnostic and prognostic markers, as well as identification of therapeutic targets.^43^

It is likely that experimental and analytic approaches to RRBS derivatives will be further refined in the near future. In order to decrease variability in coverage and increase both read depth and overlap between libraries, researchers may select a narrower range of fragments to enrich for certain CpG contexts, and/or multiplex fewer samples per lane to return more reads per library. Experiments that systematically explore fragment selection and multiplexing would help elucidate these possibilities. Improvements to genomic tiling and other regional-based analyses could also augment our ability to detect differences between samples, such as those associated with diseases or environmental exposures. Moreover, regional changes in DNAm associated with a variable interest may have more functional relevance than locus-specific changes, since gene expression is not always correlated with DNAm at individual CpG sites. Analysis pipelines that take advantage of the large number of overlapping genomic regions between any given two RRBS libraries could yield more powerful results; perhaps we can even adapt analytic approaches that have already been developed in other fields.

## Methods

Details available as Supplement.

### Microarray data

Microarray data will be posted to GEO in accordance with MIAME.

## Electronic supplementary material

Supplemental Materials

Supplementary Figure S1

Supplementary Table S1

Supplementary Table S2

Supplementary Table S3

Supplementary Table S4
