# Supplementary material for: Empirical comparison of reduced representation bisulfite sequencing and Infinium BeadChip reproducibility and coverage of DNA methylation in humans
Source: NPJ Genom Med. 2017 Apr 19;2:13. doi: 10.1038/s41525-017-0012-9 (PMC5642382; doi:10.1038/s41525-017-0012-9)

**Supplemental Materials**

The number of reads at specific CpG loci for each of the twelve rmRRBS libraries included in Figures 1-3, 5-6 can be viewed in Genome Browser. This data hub also includes the locations of CpG loci covered on both the 450K and 850K arrays. This data can be viewed via the following steps.

1. Go to UCSC Genome Browser Track Hub

(<https://genome.ucsc.edu/cgi-bin/hgHubConnect>)

2. Under the My Hubs tab, enter the following URL and click “Add Hub”: <http://www.epicenteredresearch.com/amlbinder/RRBS/hub.txt>


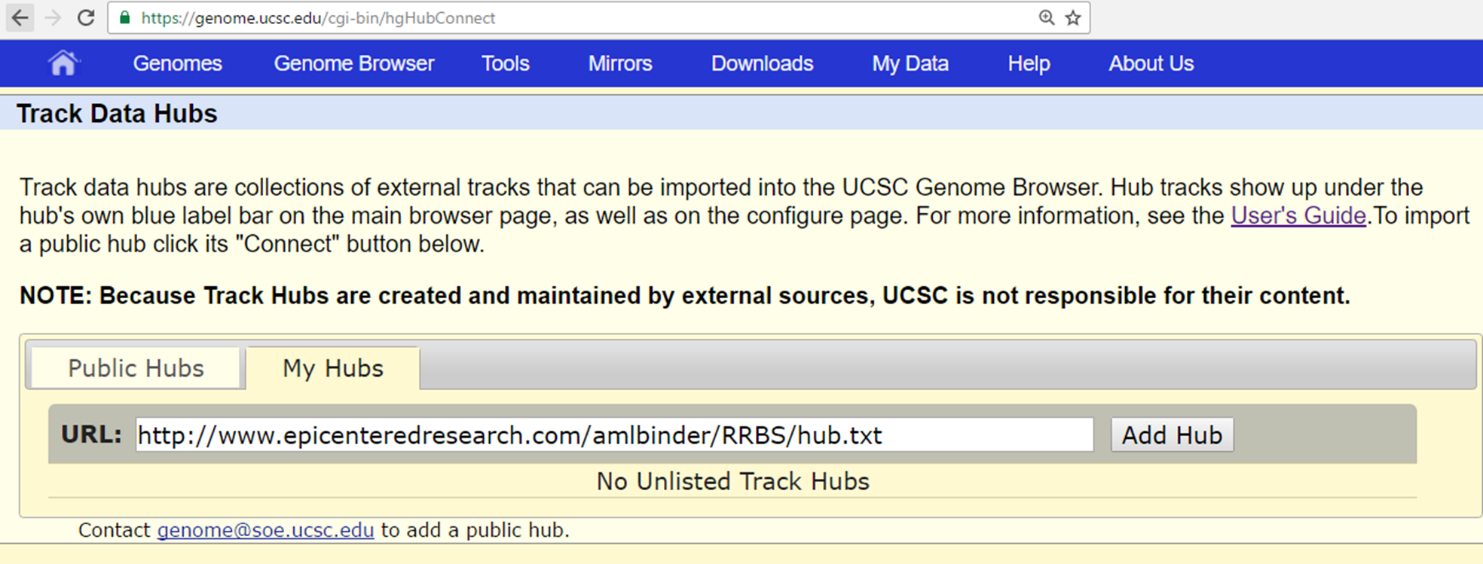


3. Enter gene or region of interest or just hit “Go”


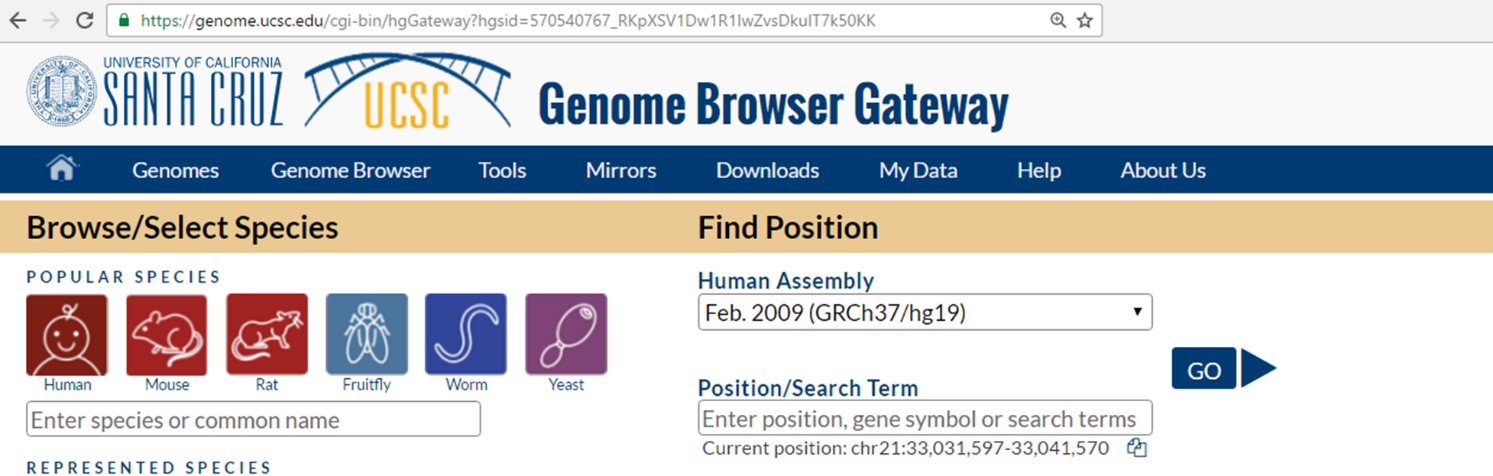


4. You can now browse the number of reads at each CpG locus for each RRBS library and see Infinium 450K and 850K coverage in the UCSC Genome Browser.


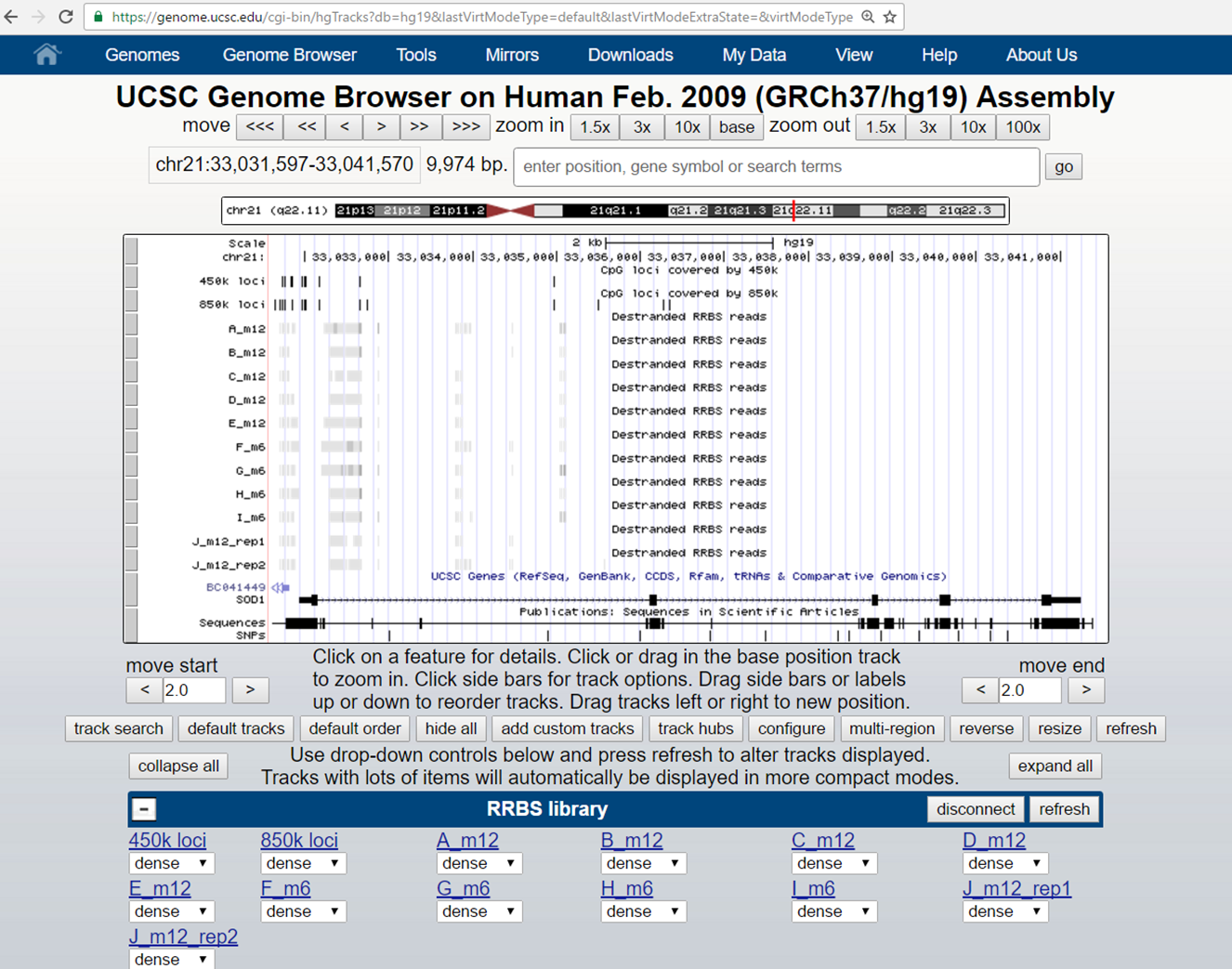

Supplement: Supplementary file 1 — Supplemental Materials [file 41525_2017_12_MOESM1_ESM.docx]
