# Supplementary material for: Empirical comparison of reduced representation bisulfite sequencing and Infinium BeadChip reproducibility and coverage of DNA methylation in humans
Source: NPJ Genom Med. 2017 Apr 19;2:13. doi: 10.1038/s41525-017-0012-9 (PMC5642382; doi:10.1038/s41525-017-0012-9)
Supplement: Supplementary file 2 — Supplementary Figure S1 [file 41525_2017_12_MOESM2_ESM.pdf]

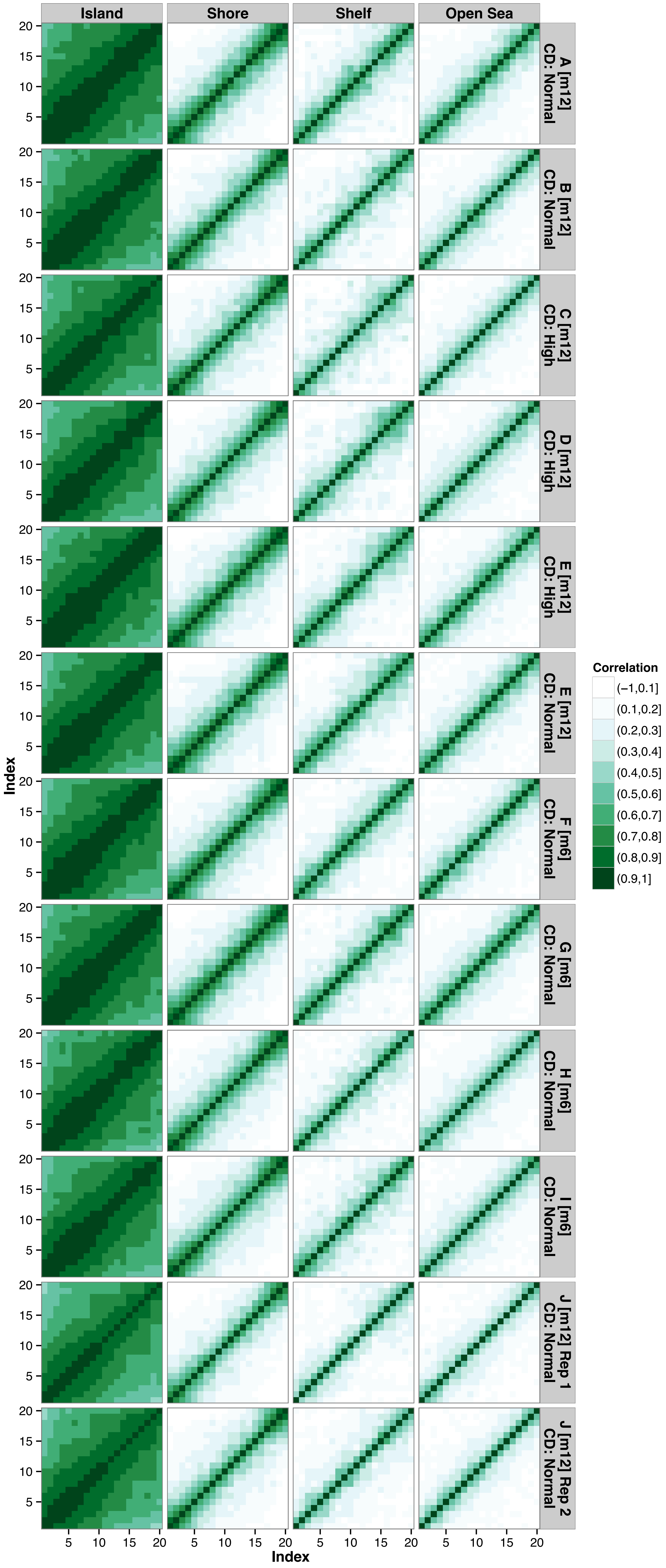

Island
Shore
Shelf
Open Sea
A [m12] CD: Normal
B [m12] CD: Normal
C [m12] CD: High
D [m12] CD: High
E [m12] CD: High
E [m12] CD: Normal
F [m6] CD: Normal
G [m6] CD: Normal
H [m6] CD: Normal
I [m6] CD: Normal
J [m12] Rep 1 CD: Normal
J [m12] Rep 2 CD: Normal
Correlation
(−1,0.1]
(0.1,0.2]
(0.2,0.3]
(0.3,0.4]
(0.4,0.5]
(0.5,0.6]
(0.6,0.7]
(0.7,0.8]
(0.8,0.9]
(0.9,1]
Index
Index
5
10
15
20

Supplementary Figure S1: Correlation in DNA methylation by distance between CpG loci, stratified by resort context. Genome-wide DNA methylation data from rmRRBS libraries was grouped into genomic tiles of up to 2,000 base-pairs (2kb) in length, but restricted to one CpG resort context (i.e. a CpG island, shore, shelf or open sea) which were then subsetted to those with at least 10 reads across the tile with a minimum tile size of 100 base-pairs. These tiles were then divided into 100 base-pair blocks, resulting in up to 20 indices (each index corresponding to a different block) within each tile. DNA methylation was averaged within each block, and the Pearson correlation between each index-pair was calculated. The average correlation for index-pairs is displayed, stratified by CpG resort context of the tile in columns and study participant in rows, such that increased green saturation indicates a higher correlation.
