# Supplementary material for: Empirical comparison of reduced representation bisulfite sequencing and Infinium BeadChip reproducibility and coverage of DNA methylation in humans
Source: NPJ Genom Med. 2017 Apr 19;2:13. doi: 10.1038/s41525-017-0012-9 (PMC5642382; doi:10.1038/s41525-017-0012-9)
Supplement: Supplementary file 3 — Supplementary Table S1 [file 41525_2017_12_MOESM3_ESM.pdf]

Supplementary Table S1A: Summary of rapid multiplexed RRBS workflow for all samples used in the study.

Participant(s)	***A, B, E***	***C, D, E***	***F, G, H, I***	***J***					
Starting gDNA Input	200ng	200ng	200ng	60ng (x12)	60ng (x12)	100ng (x12)	100ng (x12)	200ng (x12)	200ng (x12)
Library Preparation	Method 1	Method 1	Method 1	Method 1	Method 2	Method 1	Method 2	Method 1	Method 2
Replicates	None	None	None	n = 12	n = 12	n = 12	n = 12	n = 12	n = 12
Multiplexity	12-plex	12-plex	6-plex	12-plex	12-plex	12-plex	12-plex	12-plex	12-plex
Cluster Density	Normal	High	Normal	Normal	Normal	Normal	Normal	Normal	Normal
