# Supplementary material for: Empirical comparison of reduced representation bisulfite sequencing and Infinium BeadChip reproducibility and coverage of DNA methylation in humans
Source: NPJ Genom Med. 2017 Apr 19;2:13. doi: 10.1038/s41525-017-0012-9 (PMC5642382; doi:10.1038/s41525-017-0012-9)
Supplement: Supplementary file 4 — Supplementary Table S2 [file 41525_2017_12_MOESM4_ESM.pdf]

Figure 1a rmRRBS CpGs by context

coverage	annotation	median	min	max
1	Island	2033572.5	1552755	2303238
1	Shore	703281	548149	828290
1	Shelf	217635	168521	261842
1	Open Sea	1310822	1002310	1578560
2	Island	1817582.5	1198971	2041582
2	Shore	602903	424920	749456
2	Shelf	183646	129493	233966
2	Open Sea	1086175	764292	1396408
3	Island	1588989	955372	1896942
3	Shore	535340	342222	691770
3	Shelf	162414	103510	214840
3	Open Sea	956580	609413	1279735
4	Island	1442771.5	772086	1778221
4	Shore	479379.5	280627	645003
4	Shelf	144683	84449	199668
4	Open Sea	852143.5	495734	1187784
5	Island	1308477	631695	1677030
5	Shore	429742	232319	604216
5	Shelf	128745	69141	186513
5	Open Sea	762381	408758	1110562
6	Island	1161169	521775	1587958
6	Shore	387352.5	193622	567747
6	Shelf	114998.5	57262	174849
6	Open Sea	679567	340336	1041433
7	Island	1057177.5	432625	1505976
7	Shore	350093.5	162331	534655
7	Shelf	103067.5	47794	164151
7	Open Sea	607535	285371	977692
8	Island	968128	328362	1428864
8	Shore	316619.5	125387	502868
8	Shelf	92384	36713	153968
8	Open Sea	543893	233149	917399
9	Island	886693	232502	1356340
9	Shore	286784	95086	473460
9	Shelf	82814.5	27590	144405
9	Open Sea	487022	177102	859883
10	Island	811936	164749	1287650
10	Shore	259622.5	72326	445930
10	Shelf	74359.5	21022	135677
10	Open Sea	436423	134709	812488

Figure 1a 450k CpGs by context

annotation	value
Island	209409
Open Sea	114786
Shelf	39789
Shore	118439

Figure 1a 850k CpGs by context

annotation	value
Island	225957
Open Sea	395423
Shelf	64681
Shore	178876

Figure 1a In Silico RRBS CpGs

annotation	value
Island	1209613
Open Sea	2721493
Shelf	393584
Shore	973830

Figure 1b rmRRBS Number Contexts

coverage	annotation	median	min	max
1	Island	46702.5	44217	48423
1	Shore	64914.5	58977	68943
1	Shelf	26807	23602	29369
1	Open Sea	28408.5	27608	28971
2	Island	45042	41128	47252
2	Shore	60648.5	52892	66084
2	Shelf	24195.5	20597	27502
2	Open Sea	27758.5	26736	28444
3	Island	43669.5	38367	46326
3	Shore	57867.5	47898	63993
3	Shelf	22786	18331	26203
3	Open Sea	27371	25965	28190
4	Island	42429.5	35722	45483
4	Shore	55369.5	43694	62137
4	Shelf	21537	16414	25243
4	Open Sea	27027	25146	27957
5	Island	41173.5	33246	44705
5	Shore	53007.5	39781	60564
5	Shelf	20409.5	14680	24397
5	Open Sea	26674	24339	27759
6	Island	39895	30820	43972
6	Shore	50834.5	36244	59111
6	Shelf	19335	13106	23666
6	Open Sea	26308.5	23512	27574
7	Island	38570.5	28506	43238
7	Shore	48648	32854	57714
7	Shelf	18256	11721	22951
7	Open Sea	25920.5	22655	27410
8	Island	37214.5	26269	42536
8	Shore	46486.5	29577	56366
8	Shelf	17204	10193	22285
8	Open Sea	25514.5	21653	27202
9	Island	35921.5	24147	41838
9	Shore	44371.5	25950	55044
9	Shelf	16189	8538	21628
9	Open Sea	25077.5	20145	27022
10	Island	34738.5	22047	41181
10	Shore	42275.5	22056	53733
10	Shelf	15222	7145	20941
10	Open Sea	24616.5	18679	26834

Figure 1b 450k Number Contexts

annotation	all450knumbercovered
Island	49220
Open Sea	19450
Shelf	31778
Shore	56574

Figure 1b In Silico RRBS Number Contexts

annotation	RRBSnumbercovered
Island	54515
Open Sea	30706
Shelf	41942
Shore	82170

Figure 1c proportion of 450k Contexts

coverage	annotation	median	min	max
1	Island	0.801097115	0.7667411621	0.8252945957
1	Shore	0.725289002	0.6687524304	0.761374483
1	Shelf	0.4500440556	0.4005601359	0.4915979609
1	Open Sea	0.9627763496	0.9500771208	0.9751156812
2	Island	0.7789618041	0.7240146282	0.8096911824
2	Shore	0.6860483614	0.6102273129	0.7368579206
2	Shelf	0.409859022	0.3520674681	0.4632764806
2	Open Sea	0.9545758355	0.9337275064	0.9635475578
3	Island	0.7594981715	0.6851279967	0.7971556278
3	Shore	0.6603740234	0.5600098985	0.7186340015
3	Shelf	0.3877210649	0.3158159733	0.4424759267
3	Open Sea	0.9460411311	0.9183547558	0.9596915167
4	Island	0.7425233645	0.6453880536	0.7859609915
4	Shore	0.635848623	0.5161558313	0.7014352883
4	Shelf	0.3673925357	0.2851343697	0.4268047077
4	Open Sea	0.9397686375	0.8995886889	0.9558868895
5	Island	0.7247866721	0.6081064608	0.775172694
5	Shore	0.6127019479	0.4744228798	0.6866228303
5	Shelf	0.3491251809	0.256466738	0.412486626
5	Open Sea	0.9335732648	0.8812339332	0.9522879177
6	Island	0.7064099959	0.5691385616	0.764973588
6	Shore	0.5913758971	0.4359246297	0.6727825503
6	Shelf	0.3314242558	0.2294354585	0.4015356536
6	Open Sea	0.9261953728	0.8609254499	0.9494087404
7	Island	0.6872612759	0.5302112962	0.7556481105
7	Shore	0.5688920706	0.3975677873	0.6595079012
7	Shelf	0.3136131915	0.2055195418	0.3901441249
7	Open Sea	0.9180205656	0.8386118252	0.9470437018
8	Island	0.6677976432	0.4922998781	0.7457131247
8	Shore	0.5471417966	0.3598472797	0.6465690953
8	Shelf	0.2967304424	0.1790546919	0.3802945434
8	Open Sea	0.9089974293	0.8133676093	0.9428791774
9	Island	0.6504672897	0.4549370175	0.7358187729
9	Shore	0.5255064164	0.3142609679	0.6335065578
9	Shelf	0.2801309082	0.1511108314	0.3696897224
9	Open Sea	0.8992287918	0.7719794344	0.9397943445
10	Island	0.632852499	0.4173506705	0.7263917107
10	Shore	0.5033142433	0.268268109	0.6209212713
10	Shelf	0.264145006	0.1263767386	0.3590849015
10	Open Sea	0.8887403599	0.7270951157	0.9364524422

Figure 1d proportion of 850k Contexts

coverage	annotation	median	min	max
1	Island	0.7991329189	0.7644396162	0.8233246826
1	Shore	0.7169158837	0.6594266764	0.7540295475
1	Shelf	0.4578452858	0.407919595	0.49915615
1	Open Sea	0.9390413465	0.9206000732	0.9542261251
2	Island	0.7764801738	0.721528155	0.8077333172
2	Shore	0.6768444796	0.5999581734	0.7287543134
2	Shelf	0.4174412454	0.3596564145	0.4706667674
2	Open Sea	0.9248444932	0.8990852543	0.9405415295
3	Island	0.7569457018	0.6828414509	0.7951998712
3	Shore	0.6504040751	0.549034253	0.7096186308
3	Shelf	0.3949721655	0.3225522053	0.449860198
3	Open Sea	0.9149286498	0.8783754116	0.9346139773
4	Island	0.7398153178	0.6430683807	0.7840143239
4	Shore	0.6257039571	0.5049967883	0.6921709514
4	Shelf	0.374619008	0.2908637497	0.4340663493
4	Open Sea	0.9059458471	0.8553969996	0.9289791438
5	Island	0.7221920454	0.6059307542	0.7731908987
5	Shore	0.6021017881	0.4630207789	0.6772029936
5	Shelf	0.3562180408	0.2627522104	0.4200609587
5	Open Sea	0.8970911087	0.8320892792	0.924039517
6	Island	0.70364335	0.5671032249	0.7628503028
6	Shore	0.580501322	0.4248539803	0.6632657634
6	Shelf	0.3387868712	0.2356230636	0.4085493337
6	Open Sea	0.8873582144	0.8073545554	0.9199780461
7	Island	0.6844106464	0.5281751061	0.7535357193
7	Shore	0.5579448187	0.386941129	0.6495376664
7	Shelf	0.3212675382	0.2117181793	0.3971132774
7	Open Sea	0.8766373948	0.7807903403	0.9161361142
8	Island	0.6655300058	0.4904339429	0.7435974812
8	Shore	0.5358887412	0.3498199961	0.6363921545
8	Shelf	0.3038237739	0.1840852414	0.3865840449
8	Open Sea	0.8650750091	0.7501646542	0.910428101
9	Island	0.6481179713	0.4530347838	0.7336391253
9	Shore	0.5139745754	0.3054389555	0.6231719523
9	Shelf	0.2872742386	0.1551172574	0.3761303811
9	Open Sea	0.8529088913	0.7011708745	0.9057080132
10	Island	0.63029352	0.4157563321	0.7241635987
10	Shore	0.4917467099	0.2604305155	0.6102953259
10	Shelf	0.2713292526	0.1297513791	0.3651477367
10	Open Sea	0.839571899	0.6527991218	0.9008781559

Figure 2a - rmRRBS CpGs by Gene

coverage	annotation	median	min	max
1	Protein Coding	3061168.5	2352815	3460294
1	Cancer Associated Genes	144261	110349	164584
1	Mitochondrial Related	156240.5	121786	177496
1	miRNA	23064.5	17765	26637
2	Protein Coding	2659094	1819888	3151994
2	Cancer Associated Genes	124872.5	84833	150023
2	Mitochondrial Related	136567.5	94920	160983
2	miRNA	19737	13537	24181
3	Protein Coding	2383546.5	1456377	2919485
3	Cancer Associated Genes	111864.5	67560	138323
3	Mitochondrial Related	122817.5	76883	149541
3	miRNA	17496	10596	22322
4	Protein Coding	2146781.5	1182927	2731218
4	Cancer Associated Genes	101192	54756	128898
4	Mitochondrial Related	109749	62143	140086
4	miRNA	15770.5	8582	20591
5	Protein Coding	1907025	971920	2569471
5	Cancer Associated Genes	89831	44717	120889
5	Mitochondrial Related	97645.5	51342	132100
5	miRNA	14082.5	6984	19262
6	Protein Coding	1703282.5	805671	2426435
6	Cancer Associated Genes	79548	36903	113797
6	Mitochondrial Related	88099.5	42802	124899
6	miRNA	12388.5	5901	18019
7	Protein Coding	1549418.5	671134	2295350
7	Cancer Associated Genes	71700.5	30762	107642
7	Mitochondrial Related	80166	35837	118059
7	miRNA	11216	4961	16946
8	Protein Coding	1410190	507275	2170211
8	Cancer Associated Genes	65139	24777	101345
8	Mitochondrial Related	72952	27178	111593
8	miRNA	10154.5	3542	15928
9	Protein Coding	1284387.5	368034	2052948
9	Cancer Associated Genes	59309.5	17777	95553
9	Mitochondrial Related	66365	20161	105551
9	miRNA	9198	2527	14986
10	Protein Coding	1173097	267770	1941512
10	Cancer Associated Genes	53957.5	12962	89925
10	Mitochondrial Related	60327.5	14662	100126
10	miRNA	8308	1898	14080

Figure 2a 450k CpGs by Gene

annotation	value
Cancer Associated Genes	16550
miRNA	2712
Mitochondrial Related	16972
Protein Coding	389989

Figure 2a In Silico RRBS CpGs by Gene

annotation	value
Cancer Associated Genes	149053
miRNA	27218
Mitochondrial Related	182599
Protein Coding	3185669

Figure 2b 450k Number Genes

annotation	all450knumbercovered
Cancer Associated Genes	541
miRNA	599
Mitochondrial Related	1448
Protein Coding	18603

Figure 2b In Silico RRBS Number Genes

annotation	RRBSnumbercovered
Cancer Associated Genes	547
miRNA	1297
Mitochondrial Related	1442
Protein Coding	18458

Figure 2b - rmRRBS Number Genes

coverage	annotation	median	min	max
1	Protein Coding	17719	17560	18003
1	Cancer Associated Genes	536	533	541
1	Mitochondrial Related	1416	1405	1428
1	miRNA	993.5	905	1077
2	Protein Coding	17608.5	17374	17744
2	Cancer Associated Genes	535	532	539
2	Mitochondrial Related	1410	1393	1420
2	miRNA	926	815	1003
3	Protein Coding	17529.5	17214	17678
3	Cancer Associated Genes	534.5	529	538
3	Mitochondrial Related	1404	1386	1417
3	miRNA	877	738	976
4	Protein Coding	17448	17070	17622
4	Cancer Associated Genes	534	529	538
4	Mitochondrial Related	1399	1369	1412
4	miRNA	837	679	940
5	Protein Coding	17389	16900	17578
5	Cancer Associated Genes	533	527	536
5	Mitochondrial Related	1396	1345	1407
5	miRNA	805	633	918
6	Protein Coding	17331	16701	17536
6	Cancer Associated Genes	532.5	523	536
6	Mitochondrial Related	1389	1332	1405
6	miRNA	777	591	895
7	Protein Coding	17265	16443	17503
7	Cancer Associated Genes	532	519	536
7	Mitochondrial Related	1380.5	1293	1404
7	miRNA	748	540	876
8	Protein Coding	17180.5	16199	17463
8	Cancer Associated Genes	531	515	534
8	Mitochondrial Related	1377	1244	1402
8	miRNA	708	494	856
9	Protein Coding	17077	15888	17420
9	Cancer Associated Genes	529	512	534
9	Mitochondrial Related	1368.5	1191	1395
9	miRNA	676.5	436	839
10	Protein Coding	16957.5	15470	17381
10	Cancer Associated Genes	527.5	506	533
10	Mitochondrial Related	1363.5	1165	1392
10	miRNA	642	381	818

Figure 2c - Poportion of 450k Genes

coverage	annotation	median	min	max
1	Protein Coding	0.9473203247	0.9399021663	0.9624254153
1	Cancer Associated Genes	0.988909427	0.9833641405	0.9981515712
1	Mitochondrial Related	0.9734116022	0.966160221	0.9820441989
1	miRNA	0.745409015	0.7078464107	0.7779632721
2	Protein Coding	0.9425361501	0.9309788744	0.9490404773
2	Cancer Associated Genes	0.9870609982	0.9815157116	0.9944547135
2	Mitochondrial Related	0.9696132597	0.9578729282	0.9758287293
2	miRNA	0.7161936561	0.6577629382	0.7479131886
3	Protein Coding	0.938343278	0.9226468849	0.9459227006
3	Cancer Associated Genes	0.9861367837	0.9759704251	0.9926062847
3	Mitochondrial Related	0.9647790055	0.953038674	0.9737569061
3	miRNA	0.6961602671	0.6243739566	0.7378964942
4	Protein Coding	0.9344998119	0.9149599527	0.9431812073
4	Cancer Associated Genes	0.9852125693	0.9759704251	0.9926062847
4	Mitochondrial Related	0.9620165746	0.9412983425	0.9709944751
4	miRNA	0.6777963272	0.5893155259	0.7278797997
5	Protein Coding	0.9316776864	0.9060366608	0.9412460356
5	Cancer Associated Genes	0.9833641405	0.9722735675	0.988909427
5	Mitochondrial Related	0.9595994475	0.9247237569	0.9675414365
5	miRNA	0.6627712855	0.5659432387	0.7178631052
6	Protein Coding	0.9286674192	0.8955544805	0.9391495995
6	Cancer Associated Genes	0.9824399261	0.9648798521	0.988909427
6	Mitochondrial Related	0.9551104972	0.9157458564	0.9654696133
6	miRNA	0.6502504174	0.5375626043	0.7078464107
7	Protein Coding	0.9252808687	0.8818470139	0.9375907112
7	Cancer Associated Genes	0.9815157116	0.9574861368	0.988909427
7	Mitochondrial Related	0.9492403315	0.8888121547	0.9647790055
7	miRNA	0.6335559265	0.5025041736	0.7011686144
8	Protein Coding	0.9210073644	0.8689458689	0.9356017847
8	Cancer Associated Genes	0.9796672828	0.9500924214	0.9852125693
8	Mitochondrial Related	0.9468232044	0.8549723757	0.9633977901
8	miRNA	0.6143572621	0.4741235392	0.6978297162
9	Protein Coding	0.9155512552	0.8523356448	0.9334515938
9	Cancer Associated Genes	0.9759704251	0.9445471349	0.9852125693
9	Mitochondrial Related	0.9409530387	0.8183701657	0.9585635359
9	miRNA	0.5984974958	0.4373956594	0.6861435726
10	Protein Coding	0.909261947	0.8299736602	0.9314626673
10	Cancer Associated Genes	0.9731977819	0.9334565619	0.9833641405
10	Mitochondrial Related	0.9375	0.8011049724	0.9571823204
10	miRNA	0.5826377295	0.387312187	0.6777963272

Figure 2d - Poportion of 850k Genes

coverage	annotation	median	min	max
1	Protein Coding	0.9455503826	0.9379247605	0.9605608177
1	Cancer Associated Genes	0.988909427	0.9833641405	0.9981515712
1	Mitochondrial Related	0.9734848485	0.9662534435	0.9820936639
1	miRNA	0.6813241107	0.6353754941	0.7183794466
2	Protein Coding	0.9404666346	0.9285064483	0.947075507
2	Cancer Associated Genes	0.9870609982	0.9815157116	0.9944547135
2	Mitochondrial Related	0.9696969697	0.9579889807	0.9758953168
2	miRNA	0.645256917	0.5790513834	0.685770751
3	Protein Coding	0.9362926098	0.9200513726	0.9439717451
3	Cancer Associated Genes	0.9861367837	0.9759704251	0.9926062847
3	Mitochondrial Related	0.9648760331	0.9531680441	0.9738292011
3	miRNA	0.6195652174	0.5375494071	0.6729249012
4	Protein Coding	0.932252368	0.912398994	0.9412960882
4	Cancer Associated Genes	0.9852125693	0.9759704251	0.9926062847
4	Mitochondrial Related	0.9621212121	0.9414600551	0.9710743802
4	miRNA	0.5963438735	0.4980237154	0.6541501976
5	Protein Coding	0.9292021191	0.9033552737	0.9391555627
5	Cancer Associated Genes	0.9833641405	0.9722735675	0.988909427
5	Mitochondrial Related	0.9597107438	0.9249311295	0.967630854
5	miRNA	0.5815217391	0.4752964427	0.6373517787
6	Protein Coding	0.9261518703	0.8927061594	0.9369615241
6	Cancer Associated Genes	0.9824399261	0.9648798521	0.988909427
6	Mitochondrial Related	0.9552341598	0.9159779614	0.9655647383
6	miRNA	0.5622529644	0.4446640316	0.628458498
7	Protein Coding	0.9226735163	0.878953283	0.9353561299
7	Cancer Associated Genes	0.9815157116	0.9574861368	0.988909427
7	Mitochondrial Related	0.9493801653	0.8891184573	0.9648760331
7	miRNA	0.5434782609	0.4100790514	0.6185770751
8	Protein Coding	0.9183121956	0.8658960775	0.9332691176
8	Cancer Associated Genes	0.9796672828	0.9500924214	0.9852125693
8	Mitochondrial Related	0.946969697	0.8553719008	0.9634986226
8	miRNA	0.5197628458	0.3754940711	0.6077075099
9	Protein Coding	0.9128270991	0.8492534917	0.9310750789
9	Cancer Associated Genes	0.9759704251	0.9445471349	0.9852125693
9	Mitochondrial Related	0.9411157025	0.8188705234	0.958677686
9	miRNA	0.4985177866	0.3399209486	0.597826087
10	Protein Coding	0.9064857923	0.827152566	0.9290950928
10	Cancer Associated Genes	0.9731977819	0.9334565619	0.9833641405
10	Mitochondrial Related	0.9376721763	0.8016528926	0.9573002755
10	miRNA	0.4812252964	0.2984189723	0.5899209486

Figure 3 450k CpGs by genes and tiles

Group	NumOverlap
Cancer Associated Genes	541
miRNA	599
Mitochondrial Related	1448
Protein Coding	18603
CpG Locus	482421
200bp Tile	354806
2kb Tile	225403
