# Supplementary material for: Empirical comparison of reduced representation bisulfite sequencing and Infinium BeadChip reproducibility and coverage of DNA methylation in humans
Source: NPJ Genom Med. 2017 Apr 19;2:13. doi: 10.1038/s41525-017-0012-9 (PMC5642382; doi:10.1038/s41525-017-0012-9)
Supplement: Supplementary file 5 — Supplementary Table S3 [file 41525_2017_12_MOESM5_ESM.pdf]

Supplementary Table S3: Laboratory protocols for rapid multiplexed RRBS library preparations

Step in Protocol	Method 1		
	Reagents & Equipment	Temperatures (Celsius)	Time(s)
DNA Fragmentation	MspI enzyme system (New England BioLabs, R0106L)<br>NEB Buffer 2 (New England Biolabs)<br>Water bath	37	16-18 hours
End repairing & A-tailing	Klenow fragment system (New England Biolabs, M0212L)<br>dNTP mix (10mM dATP, 1mM dGTP, 1mM dCTP)<br>Thermal cycler	20<br>37<br>4	20 minutes<br>20 minutes<br>hold
Adapter Ligation	TruSeq indexed methylated adapters (Illumina)<br>T4 DNA Ligase Enzyme (New England BioLabs, M0202M)<br>T4 Ligation Buffer (New England Biolabs, M0202M)<br>Thermal cycler	16<br>4	16-18 hours<br>hold
Fragment selection	Agencourt AMPure XP magnetic beads (Beckman Coulter)<br>80% Ethanol<br>10 mM Tris-HCl, pH 8.0, EDTA free<br>DynaMag 96-side magnet (Life Technologies)	Room Temp.	-
Quantificaiton & Pooling	Qubit fluorometer & reagents (Invitrogen)	Room Temp.	-
Bisulfite Conversion	EZ DNA Methylation-Gold Kit (Zymo)	See manufacturer's instructions	
Amplification	PfuTurbo Cx hotstart DNA polymerase (Agilent Technologies)<br><br>10X PCR buffer (Agilent Technologies)<br>Universal PCR primers (2.5uM each; Illumina)<br>dNTP mix (25mM each dATP, dCTP, dGTP, dTTP)	95<br>Below: cycle 16 times<br>95<br>65<br>72<br>End cycling<br>72<br>4	2 minutes<br><br>30 seconds<br>30 seconds<br>30 seconds<br><br>7 minutes<br>hold
Final Cleanup	Agencourt AMPure XP magnetic beads (Beckman Coulter)<br>80% Ethanol<br>10 mM Tris-HCl, pH 8.0, EDTA free<br>DynaMag 96-side magnet (Life Technologies)	Room Temp.	-

Suppl Table S3 rmRRBS Protocols

Method 2		
Reagents & Equipment	Temperature (Celsius)	Time(s)
MspI enzyme system (New England Biolabs)<br>NEB Buffer 2 (New England Biolabs)<br>Water bath	37	16-18 hours
NEBNext Ultra End Prep Enzyme Mix (New England Biolabs E7370SL)<br>NEBNext Ultra End Repair Reaction Buffer (New England Biolabs E7370SL)<br>Thermal cycler	20<br>65<br>4	30 minutes<br>30 minutes<br>hold
TruSeq indexed methylated adapters (Illumina)<br>NEBNext Ultra Enhancer (New England Biolabs E7370SL)<br>NEBNext Ultra T4 Ligase Master Mix (New England Biolabs E7370SL)<br>NEBNext Ultra USER enzyme(New England Biolabs E7370SL)<br>Thermal cycler	20<br>37<br>4	15 minutes<br>15 minutes<br>hold
Agencourt AMPure XP magnetic beads (Beckman Coulter)<br>80% Ethanol<br>10 mM Tris-HCl, pH 8.0, EDTA free<br>DynaMag 96-side magnet (Life Technologies)	Room Temp.	-
Qubit fluorometer & reagents (Invitrogen)	Room Temp.	-
EZ DNA Methylation-Gold Kit (Zymo)	See manufacturer's instructions	
PfuTurbo Cx hotstart DNA polymerase (Agilent Technologies)<br><br>10X PCR buffer (Agilent Technologies)<br>Universal PCR primers (2.5uM each; Illumina)<br>dNTP mix (25mM each dATP, dCTP, dGTP, dTTP)	95<br>Below: cycle 16 times<br>95<br>65<br>72<br>End cycling<br>72<br>4	2 minutes<br><br>30 seconds<br>30 seconds<br>30 seconds<br><br>7 minutes<br>hold
Agencourt AMPure XP magnetic beads (Beckman Coulter)<br>80% Ethanol<br>10 mM Tris-HCl, pH 8.0, EDTA free<br>DynaMag 96-side magnet (Life Technologies)	Room Temp.	-
