# Supplementary material for: Empirical comparison of reduced representation bisulfite sequencing and Infinium BeadChip reproducibility and coverage of DNA methylation in humans
Source: NPJ Genom Med. 2017 Apr 19;2:13. doi: 10.1038/s41525-017-0012-9 (PMC5642382; doi:10.1038/s41525-017-0012-9)
Supplement: Supplementary file 6 — Supplementary Table S4 [file 41525_2017_12_MOESM6_ESM.pdf]

Supplemental Table S4. Comparison of the number of CpG loci captured by the two processing protocols (rmRRBS and NEBnext); Restricting to CpG loci with at least 10x coverage and libraries starting with 200ng gDNA. All libraries are derived from the same initial whole blood DNA sample.

	Median (Range)	
**Annotation**	**rmRRBS (n=12)**	**NEBnext (n=12)**
**All**	182,647.5 (8,774–1,215,847)	121,625 (17,050–278,598)
**CpG Island Context**		
Island	72,874 (2,731–587,614)	55,107 (5,218–140,165)
Open Sea	6,4861 (3,652–365,455)	38,756.5 (7,292–79,978)
Shelf	9,833.5 (528–59,682)	5,576.5 (1,067–11,877)
Shore	35,079 (1,863–203,096)	21,877 (3,473–46,578)
